# Clinical Characteristics and Outcomes of Nontuberculous Mycobacterial Empyema

**DOI:** 10.7759/cureus.82337

**Published:** 2025-04-15

**Authors:** Hitoshi Suzuki, Daisuke Ito, Mari Shinoda, Shin Shomura, Kentaro Inoue, Akira Shimamoto

**Affiliations:** 1 Department of Thoracic Surgery, Mie Prefectural General Medical Center, Yokkaichi, JPN; 2 Department of Thoracic and Cardiovascular Surgery, Graduate School of Medicine, Mie University, Tsu, JPN

**Keywords:** nontuberculous mycobacteriosis, pleural empyema, pneumothorax, prognosis, surgical indication

## Abstract

Introduction

The incidence of pulmonary nontuberculous mycobacterial (NTM) infections has increased in recent decades. But NTM empyema is still a rare disease. The purpose of this study was to clarify the clinical characteristics and outcomes of NTM empyema.

Methods

The medical records of 484 patients with pulmonary NTM disease (NTM empyema 15, NTM disease without empyema 469) and 367 patients with acute empyema (NTM empyema 15, other empyema 352) were retrospectively reviewed and investigated patients with empyema with other causes as well as patients with NTM empyema from January 2012 to December 2024 in our hospital. NTM empyema was defined as a positive NTM culture of pleural effusion samples and had purulent pleural effusion.

Results

Among patients with pulmonary NTM and patients with acute empyema in our study, 15 had NTM empyema (3.1%, 4.1%). Age (median 78 vs. 68, p=0.0072), male (60.0% vs. 32.4%, p=0.0256), Charlson Comorbidity Index (CCI, median 6 vs. 4, p=0.0007) and fibrocavitary form patients (60.0% vs. 21.5%, p=0.0005) were higher in the NTM empyema than in the NTM disease without empyema. Although the same long-term follow-up as NTM disease without empyema was performed, the long-term prognosis of NTM empyema was poorer than that of NTM disease without empyema. Ten out of 15 patients with NTM empyema and 52 out of 352 patients with other empyema patients were complicated with pneumothorax. Compared to patients with other empyema, patients with NTM empyema had higher incidences of pneumothorax (66.7% vs. 14.8%, p=0.00001). Surgical treatments were performed for 11 patients of 15 NTM empyema - two patients died (18.2%) - and were more frequently performed for other empyema. There were significant differences between the mortality rate for fistulous patients with NTM empyema and those for fistulous patients with other empyema (40.0% vs. 11.5%, p=0.0250). All patients with NTM empyema who died had pneumothorax. No fistulous patient with NTM empyema was cured without surgical treatment.

Conclusions

NTM empyema patients had higher rates of fibrocavitary form, were male, and had higher CCI, and the long-term prognosis of NTM empyema was poorer than that of NTM disease without empyema. In addition, this study revealed that NTM empyema has a poor prognosis and is difficult to treat without chest tube drainage or surgical treatment. Early surgical intervention should be considered for patients with fistulous NTM empyema.

## Introduction

Nontuberculous mycobacterial (NTM) infection can cause chronic and slowly progressive pulmonary disease. The increasing prevalence of pulmonary infections caused by NTM pathogens is an emerging public health concern worldwide, but the management of pulmonary NTM disease was heterogeneous and cure rates were relatively low despite the publication of the 2020 guidelines by the American Thoracic Society (ATS), European Respiratory Society (ERS), European Society of Clinical Microbiology and Infectious Diseases (ESCMID), and Infectious Diseases Society of America (IDSA) [1,2]. We compared the characteristics of NTM empyema (n=15) and NTM disease without empyema (n=469) in our hospital at the first step. Therapeutic strategies for NTM empyema were the same for other empyema.

NTM empyema is rarer than tuberculosis [3,4]. Only a few studies in the literature [5,6] have reported the clinical characteristics, pathogenesis, and prognosis of NTM empyema. Moreover, these case series of NTM empyema have described a high incidence of pneumothorax and higher mortality rates than other empyema [7]. We compared the characteristics and outcomes of NTM empyema (n=15) and other empyema (n=352) in our hospital at the second step. We therefore conducted the study to elucidate the clinical features, outcomes and surgical indication of NTM empyema. All patients were treated and followed up at one hospital. The same analysis by the same group was then performed.

This article was previously posted to the Research Square preprint server on 24 July 2024 (https://doi.org/10.21203/rs.3.rs-4608859/v1).

## Materials and methods

We retrospectively reviewed the medical records of 484 patients at our institution with pulmonary NTM disease between January 2012 and December 2024. We used the diagnostic criteria of 2020 ATS/ERS/ESCMID/IDSA [1] to diagnose NTM. In addition, we retrospectively reviewed 367 patients with empyema during the same period at our hospital. All patients underwent CT evaluation without exception. A total of 15 suffered from empyema associated with NTM infection. These 15 patients accounted for 3.1% of all NTM patients and 4.1% of all empyema patients. CT images were evaluated for cavitary lesions and radiological patterns, and all NTM patients were categorized as having the nodular-bronchiectatic (NB) or fibrocavitary (FC) form. There was an overlap in one out of 15 cases. We divided it into more advantageous groups.

Diagnostic criteria

Conventional empyema was defined as septations or loculations identified in the pleural space on CT scans or ultrasound examinations and the existence of gross pus or organisms demonstrated on a Gram stain or culture. In addition, conventional empyema was defined as septations or loculations identified in the pleural space on CT scans or ultrasound examinations and positive biochemical results when gross pus or organisms did not demonstrate on a Gram stain or culture. In terms of biochemical methods, if the concentration of sugar in pleural fluid was less than 40 mg/dL and the power of hydrogen (pH) in pleural fluid was below 7.200, empyema was diagnosed biochemically [8]. NTM empyema was diagnosed if septations or loculations were identified in the pleural space on CT scans or ultrasound examinations and if pleural effusions were positive by the truant method of fluorochrome or *Mycobacterium avium* complex polymerase chain reaction (MAC-PCR) or culture (Mycobacteria Growth Indicator Tube method: MGIT).

Therapeutic strategies for acute empyema

Antibiotics used were penicillins combined with β-lactamase inhibitors, as shown in the British Thoracic Society (BTS) guideline [8]. Drainage treatments were performed if it was determined that drainage treatments were possible based on CT or echocardiogram findings. Surgical intervention for patients where antibiotic treatments and drainage treatments have failed, according to the BTS guidelines. However, the timing of surgical intervention has not been adequately evaluated in the BTS guidelines. Therefore, the median duration from onset to surgical intervention was 18 days in the past retrospective study [7], and we sometimes lost the timing for surgical intervention. Then, we created therapeutic strategies for acute empyema as follows: early surgical intervention is recommended for (1) multiloculated empyema, (2) methicillin‐resistant *Staphylococcus aureus* empyema, or (3) no response to antibiotics treatment within three days. Exceptions: grade 4 Performance Status (PS) as described previously [7]. We shared the results of the report with doctors at our hospital and surrounding facilities. Pulmonologists or thoracic surgeons at our hospital performed the chest drainage procedure. Multiple chest tubes were frequently inserted for empyema patients with grade 4 PS. Then, the median duration from onset to surgical intervention was eight days in the past prospective study [7]. The operative technique included an endoscope that was inserted at the middle aspect of the sixth intercostal space for inspection of the pleural cavity. A transverse skin incision (approximately 5 cm) was made laterally at the largest empyema cavity level. The first step consisted of complete evacuation of the fluid component of the empyema by suction, disruption of fibrinous pleural septations, and gentle removal of minor adhesions until the empyema cavity became a single space. The next step included repeated pleural lavage with hydrogen peroxide and normal saline until the cavity was clean. Finally, 28-Fr chest tubes were placed at the apical position and on the diaphragm. A 6-Fr chest tube was inserted for irrigation. Antibiotic treatment was terminated when the serum C-reactive protein (CRP) concentration decreased to less than 5 mg/dL, and the case was discharged two days later [7]. The follow-up for NTM empyema patients was several years, and the follow-up for other empyema patients was basically one year.

Statistical analysis

All statistical analyses were performed with EZR (Saitama Medical Center, Jichi Medical University, Saitama, Japan), which is a graphical user interface for R (The R Foundation for Statistical Computing, Vienna, Austria). More precisely, it is a modified version of R commander designed to add statistical functions frequently used in biostatistics. Patient characteristics were compared using the chi-square test or Fisher’s exact test for categorical data and t-tests for continuous data. The variables in the multivariate analysis were selected from the significant variables in the univariate analysis. For the logistic regression model, we assessed collinearity using the variance inflation factor. Variables with a variance inflation factor greater than 10 were considered to be collinear, and if present, we excluded one of these variables from the analysis. A two-sided p-value < 0.05 was considered to indicate statistical significance.

## Results

Clinical characteristics of NTM empyema and NTM disease without empyema

Among the whole NTM patients of our study, the proportion of females was higher than that of males (66.7% vs. 33.3%), and predominant form and pathogen were NB (77.3%) and *M. avium* (66.9%). These results were similar to those of the other study [9]. Patients with NTM empyema were significantly older (median age, 78 vs. 68, p=0.0072), be male (60.0% vs. 32.4%, p=0.0256), and have higher Charlson Comorbidity Index (CCI) (median 6 vs. 4, p=0.0007). Patients with NTM empyema were also more likely to be immunocompromised (33.3% vs. 8.5%, p=0.0014) and have the FC form (60.0% vs. 21.5%, p=0.0005) than were the patients with NTM disease without empyema. An immunocompromised patient is defined as having one or more of the following: systemic steroids (≥10 mg), anti-rheumatic drugs, anti-cancer chemotherapy, radiation therapy, or solid organ transplantation. Hospital mortality was 26.7%. The median follow-up period after discharge was 19 months, and 54.5% of discharged patients died during the follow-up period. This was significantly higher than the 9.6% of NTM disease without empyema. These factors were statistically compared between the two groups, as can be seen in Table 1.

**Table 1:** Clinical characteristics of NTM empyema and NTM disease without empyema. Data are expressed as numbers (%) for categorical and ordinal variables and as medians (interquartile range) for continuous variables. Categorical variables were analyzed using the chi-square test or Fisher’s exact test. Continuous variables were analyzed using the t-test. A two-sided p-value < 0.05 was considered to indicate statistical significance. * indicates a statistically significant difference in the parameter between the two groups, p < 0.05. NTM: nontuberculous mycobacteria

Valuables	NTM empyema	NTM disease without empyema	p-value
Total patients, n	15	469	-
Age (years), median (range)	78 (50-91)	68 (26-96)	0.0072*
Sex			
Male, n (%)	9 (60.0)	152 (32.4)	0.0256*
Female, n (%)	6 (40.0)	317 (67.6)	
Charlson Comorbidity Index, median (range)	6 (2-14)	4 (0-10)	0.0007*
Immunocompromised cases, n (%)	5 (33.3)	40 (8.5)	0.0014*
Fibrocavitary form, n (%)	9 (60.0)	101 (21.5)	0.0005*
Nodular-bronchiectatic, n (%)	6 (40.0)	368 (78.5)	
Follow-up period (months after discharge vs. after diagnosis), median (range)	19 (4-141)	30 (1-106)	0.5436
				Mortality during follow-up, n (%)	6 (54.5)	45 (9.6)	0.0003*
Pathogens			
*Mycobacterium avium*, n (%)	11 (73.3)	313 (66.7)	0.2914
*M. intracellulare*, n (%)	2 (13.3)	97 (20.7)	
*M. kansasii*, n (%)	0 (0.0)	28 (6.0)	
*M. abscessus*, n (%)	2 (13.3)	14 (3.0)	
Others, n (%)	0 (0.0)	17 (3.6)	

**Figure 1:**
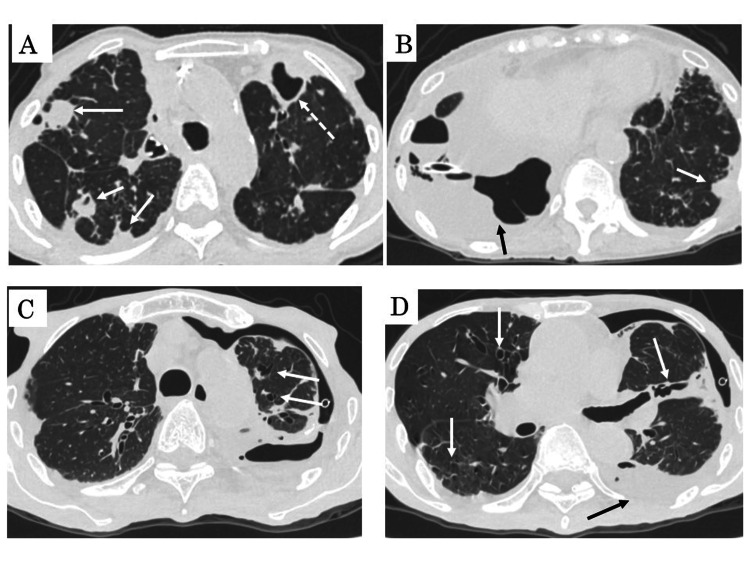
Representative figures of CT findings (1-mm thick slices) of patients with NTM empyema. (A, B) (fibrocavitary form): A 86-year-old female with NTM (*Mycobacterium avium*) empyema. Computed tomography images show consolidation (white arrows), cavity (white broken arrow), and pleural effusion (black arrow). (C, D) (nodular-bronchiectatic form): A 91-year-old male with NTM (*M. abscessus*) empyema. Computed tomography images show multiple centrilobular nodules with bronchiectasis (white arrows) in bilateral upper lobes, pneumothorax and pleural effusion (black arrow) in the left lung field. NTM: nontuberculous mycobacteria

Representative figures of CT findings (1-mm thick slices) of patients with NTM empyema are shown in Figure 1.

Clinical characteristics and outcomes of patients with NTM empyema and other empyema

According to the clinical background, age, CCI, PS, and rates of immunocompromised patients, pneumothorax, lung abscess and lung cavitation were significantly different between the two groups. According to the results of the blood test and pleural fluid test, only the pleural adenosine deaminase (ADA) level was significantly different between the two groups. For treatment options, surgical methods were significantly different between the two groups. In terms of outcomes, the rates of recurrence, fistula-related mortality within each group, and overall mortality were significantly different between the two groups. In addition, all patients with NTM empyema who died had pneumothorax, and no fistulous patient with NTM empyema was cured without surgical treatment. These factors were statistically compared between the two groups, as can be seen in Table 2.

**Table 2:** Clinical characteristics and outcomes of patients with NTM empyema and other empyema. Data are expressed as numbers (%) for categorical and ordinal variables and as medians (interquartile range) for continuous variables. Categorical variables were analyzed using the chi-square test or Fisher’s exact test. Continuous variables were analyzed using the t-test. A two-sided p-value < 0.05 was considered to indicate statistical significance. * indicates a statistically significant difference in the parameter between the two groups, p < 0.05. NTM: nontuberculous mycobacteria; pH: power of hydrogen; WBC: white blood cells; CRP: C-reactive protein; LDH: lactate dehydrogenase; ADA: adenosine deaminase

Valuables	NTM empyema	Other empyema	p-value
Total patients, n	15	352	-
Age (years), median (range)	78 (50-91)	72 (15-93)	0.0237*
Sex			
Male, n (%)	9 (60.0)	282 (80.1)	0.0597
Female, n (%)	6 (40.0)	70 (19.9)	
Charlson Comorbidity Index, median (range)	6 (2-14)	5 (0-14)	0.0026*
Performance Status, median (range)	2 (0-4)	1 (0-4)	0.0162*
Immunocompromised patients, n (%)	5 (35.3)	41 (11.6)	0.0130*
Serum WBC, /mL	12900 (4100-34800)	14300 (2900-57500)	0.1010
Serum CRP, mg/dL	19.9 (5.50-30.13)	18.5 (4.09-54.40)	0.4741
Pleural pH	7.154 (6.500-7.766)	7.224 (6.000-7.766)	0.9270
Pleural LDH, IU/L	1937 (254-38120)	1067 (25-73200)	0.4026
Pleural total protein, g/dL	4.3 (3.1-5.2)	4.6 (0.6-7.5)	0.2462
Pleural glucose, mg/dL	56.5 (0-128)	39.0 (0-547)	0.9410
Pleural ADA, U/L	90.2 (16-182)	35.0 (3.3-440)	0.0289*
Pneumothorax, n (%)	10 (66.7)	52 (14.8)	0.00001*
Mortality of fistulous patients out of each group, n (%)	4 (40.0)	6 (12.0)	0.0250*
Cured without operation, n (%)	0 (0.0)	29 (58.0)	0.0031*
Lung abscess, n (%)	4 (26.7)	21 (6.0)	0.0018*
Lung cavitation, n (%)	11 (73.3)	135 (38.7)	0.0067*
Surgical treatment, n (%)	11 (73.3)	157 (44.6)	0.0288*
Decortication (D), n (%)	4 (36.4)	116 (73.9)	0.0209*
D+lung plication, n (%)	5 (45.5)	23 (14.6)	0.0080*
D+segmentectomy, n (%)	1 (9.1)	0 (0.0)	0.4006
D+lobectomy, n (%)	1 (9.1)	11 (7.0)	
D+endobronchial occlusion, n (%)	0 (0.0)	1 (0.6)	
Open window thoracotomy, n (%)	0 (0.0)	6 (3.8)	
Postoperative mortality, n (%)	2 (18.2)	7 (4.7)	0.0507
Recurrence, n (%)	1 (6.7)	3 (0.9)	0.0357*
Total mortality, n (%)	4 (26.7)	33 (9.4)	0.0294*

Multivariate analysis showed significant differences in the presence or absence of fistula and lung abscess. These factors were statistically compared between the two groups, as can be seen in Table 3.

**Table 3:** Results of multivariate and binary logistic regression analysis. Patients with missing values were excluded (list-wise deletion) only if missing values were found in the target variables, and multivariate logistic regression was performed, including confounding factors such as age, immunosuppression status, CCI, and PS to evaluate the independent association of each factor. CI: confidence interval; ADA: adenosine deaminase; CCI: Charlson Comorbidity Index; PS: Performance Status

Valuables	Odds ratio	95% CI	p-value
Age	1.042	0.942-1.152	0.4269
Charlson Comorbidity Index	1.819	0.926-3.571	0.0822
Performance Status	0.9336	0.4756-1.8324	0.8416
Immunocompromised	2.1617	0.2303-20.2869	0.4998
Pleural ADA	1.0088	0.9992-1.0185	0.0710
Pneumothorax	29.257	2.9856-286.6941	0.0037
Lung abscess	21.913	2.0436-234.9682	0.0108
Lung cavitation	3.8726	0.5165-23.0328	0.1877

## Discussion

NTM empyema is a rare condition that has been reported in case reports [10] and retrospective studies [5,6]. Previous studies [11,12] reported that the frequency of pleural effusion in patients with pulmonary NTM was 1.4-3.4%. In our study, 15 of 469 (3.1%) patients with pulmonary NTM infection developed empyema with NTM infection isolated from pleural effusion. The most common pathogen causing pleuritis was *M. avium*, followed by* M. kansasii* and *M. intracellulare,* according to previous reports [13]. The present study showed that NTM empyema was more common in males than in women, and FC disease was seen more frequently in NTM empyema patients than in NTM patients without empyema. McShane and Glassroth [14] reported that FC disease is more common in older male smokers, while NB disease more frequently occurs in middle-aged or older women with no smoking history. Furthermore, Kim et al. [15] reported that the bacterial burden in the FC form is much higher than that in the NB form, which can be evidence that the disease course of the FC form may be more aggressive than the NB form. These factors may overlap with the characteristics often seen in NTM empyema patients.

The present study showed that 11 of 15 patients (73.3%) with NTM empyema had cavitary lesions, and the percentage was higher than patients with other empyema. Moreover, Yagi et al. [6] reported that patients with MAC pleuritis tended to have more cavitary lesions than sex- and age-matched patients presenting with MAC lung disease without pleuritis. Cavitary lesions are formed as a result of drainage into the airway in parenchymal caseous necrosis in patients with advanced NTM lung disease, and their presence is reported as a clinically important factor.

Previous reports revealed that 40-70% of patients with NTM pleuritis were complicated by pneumothorax, and the mechanism of NTM pleuritis was primarily suspected to be the perforation of pulmonary NTM disease or the spread of inflammation to the pleura [5,6,12,16].

Multivariate analysis of our study showed that NTM empyema patients had significantly higher incidences of pneumothorax and lung abscess than did other empyema patients. In our study, no patient with fistulous NTM empyema was cured without surgical treatment. In our study and the previous report [7], 15-18% of patients with other empyema were complicated by pneumothorax, and other empyema patients who were cured of pneumothorax without surgical treatment for lungs were seen more frequently than NTM empyema patients (55.8% vs. 0%, p=0.0031). This result may support the idea that NTM empyema can develop through leakage or perforation of pulmonary NTM and that bronchopleural fistulas are more common in NTM empyema.

Kobashi et al. [17] reported that pneumothorax occurred in 4.1% of NTM patients, and NTM pleuritis arose secondarily in one-third of the pneumothorax patients. In addition, they reported that the responses to treatment and prognoses were poor due to the presence of other complications. Ando et al. [12] reported that comorbid NTM pleuritis was difficult to treat by medical therapy alone and resulted in a poor prognosis. In addition to antimycobacterial agents, chest tube drainage and surgical procedures in the early stages should be considered to treat NTM pleuritis. Pneumothorax was a frequent complication of NTM empyema and was difficult to treat without chest tube drainage or surgical treatment. Therefore, early surgical intervention should also be considered for patients with fistulous NTM empyema.

Previous reports revealed that the hospital mortality rate is 25-29% [11-16]. The authors, including us, also emphasized the importance of immediate therapy, especially drainage and surgical treatment, using a multidisciplinary approach for treating NTM empyema. In this study, the hospital mortality rate was 26.7% (4 of 15 patients), which was comparable to that in previous reports [11,16]. Sugiura et al. [18] reported that the mortality rate was 3.8% for non-fistulous empyema and 44.4% for fistulous empyema. In our study, the mortality rate was 0% for non-fistulous NTM empyema, but the mortality rate was 40.0% for fistulous NTM empyema, although the mortality rate was 12.0% for other fistulous empyema in our study. Ikeda et al. [19] reported that we should select an appropriate treatment, including surgery against NTM-associated pneumothorax without losing an opportunity because of its intractability and exhausting effect. Our study and the previous reports [6,7] revealed that NTM empyema patients had a poorer prognosis than did other empyema patients, and the causative factors might be older age, poorer PS, higher CCI, and higher incidences of lung cavitation and pneumothorax. Therefore, early surgical intervention should be considered for patients with fistulous NTM empyema. However, the effect of early detection and surgical intervention for patients with fistulous NTM empyema is unclear because this study is a retrospective study. In addition to the therapeutic strategies for acute empyema, a prospective study should be conducted to consider early surgical intervention for patients with fistulous NTM empyema. A multicenter prospective study is necessary because NTM empyema is a rare condition, and the sample size is small.

Limitations

This study has several limitations. First, this study involved an analysis of all empyema patients from a single institution, but 53.8% of NTM empyema patients and 46.2% of other empyema patients were transferred from surrounding hospitals due to failed therapy before they were hospitalized at our hospital. Therefore, selection bias was inevitable. Second, because NTM disease without empyema was confirmed in patients from a single institution, these patients might not be representative of national populations. Finally, since NTM empyema is a rare condition, the number of patients examined in our study was small. The size of the sample of patients with NTM empyema was much smaller than that of patients without NTM empyema, and the statistical analysis may not be valid. However, additional large-sample studies are needed to confirm our results.

## Conclusions

This study revealed that NTM empyema had a poor prognosis compared with other empyema and was difficult to treat without chest tube drainage or surgical treatment. Early surgical intervention should be considered for patients with fistulous NTM empyema. A prospective study should be conducted to consider early surgical intervention for patients with fistulous NTM empyema. A multicenter prospective study is necessary because NTM empyema is a rare condition, and the sample size is small.

## References

[1] Daley CL, Iaccarino JM, Lange C (2020). Treatment of nontuberculous mycobacterial pulmonary disease: an official ATS/ERS/ESCMID/IDSA clinical practice guideline. Clin Infect Dis.

[2] Abate G, Stapleton JT, Rouphael N (2021). Variability in the management of adults with pulmonary nontuberculous mycobacterial disease. Clin Infect Dis.

[3] Bachar K, Shulimzon T, Segel MJ (2022). Nontuberculous mycobacteria infections of the pleura: a systematic review. Respir Med.

[4] Light RW (2010). Update on tuberculous pleural effusion. Respirology.

[5] Wen P, Wei M, Xu YR, Dong L (2020). Clinical relevance and characteristics of nontuberculous mycobacterial pleuritis. Jpn J Infect Dis.

[6] Yagi K, Ito A, Fujiwara K (2021). Clinical features and prognosis of nontuberculous mycobacterial pleuritis: a multicenter retrospective study. Ann Am Thorac Soc.

[7] Suzuki H, Shomura S, Sawada Y, Shimamoto A, Kondo C, Takao M, Shimpo H (2019). Therapeutic strategy for acute pleural empyema: comparison between retrospective study and prospective study. Gen Thorac Cardiovasc Surg.

[8] Davies HE, Davies RJ, Davies CW (2010). Management of pleural infection in adults: British Thoracic Society Pleural Disease Guideline 2010. Thorax.

[9] Izumi K, Morimoto K, Hasegawa N, Uchimura K, Kawatsu L, Ato M, Mitarai S (2019). Epidemiology of adults and children treated for nontuberculous mycobacterial pulmonary disease in Japan. Ann Am Thorac Soc.

[10] Anjum S, Tahir R, Pathan SA (2015). Nontuberculous mycobacterial infection presenting as empyema and life threatening pneumothorax: a challenging situation in the emergency department. Qatar Med J.

[11] Park S, Jo KW, Lee SD, Kim WS, Shim TS (2017). Clinical characteristics and treatment outcomes of pleural effusions in patients with nontuberculous mycobacterial disease. Respir Med.

[12] Ando T, Kawashima M, Matsui H (2018). Clinical features and prognosis of nontuberculous mycobacterial pleuritis. Respiration.

[13] Ueyama M, Asakura T, Morimoto K (2016). Pneumothorax associated with nontuberculous mycobacteria: a retrospective study of 69 patients. Medicine (Baltimore).

[14] McShane PJ, Glassroth J (2015). Pulmonary disease due to nontuberculous mycobacteria: current state and new insights. Chest.

[15] Kim BG, Kang N, Kim SY (2023). The lung microbiota in nontuberculous mycobacterial pulmonary disease. PLoS One.

[16] Naito M, Maekura T, Kurahara Y (2018). Clinical features of nontuberculous mycobacterial pleurisy: a review of 12 cases. Intern Med.

[17] Kobashi Y, Mouri K, Obase Y, Kato S, Oka M (2013). Clinical analysis of patients with pulmonary nontuberculous mycobacterial disease complicated by pneumothorax. Intern Med.

[18] Sugiura Y, Nakamura M, Fujimoto H (2023). An independent prognostic factor in surgical cases of pleural empyema caused by common bacteria is the presence of a fistula. Gen Thorac Cardiovasc Surg.

[19] Ikeda M, Takahashi K, Komatsu T, Tanaka T, Kato T, Fujinaga T (2017). The frequency and treatment of pneumothorax associated with pulmonary nontuberculous mycobacterial infection. Gen Thorac Cardiovasc Surg.

